# Novel Serum Biomarkers to Differentiate Cholangiocarcinoma from Benign Biliary Tract Diseases Using a Proteomic Approach

**DOI:** 10.1155/2015/105358

**Published:** 2015-04-28

**Authors:** Tavan Janvilisri, Kawin Leelawat, Sittiruk Roytrakul, Atchara Paemanee, Rutaiwan Tohtong

**Affiliations:** ^1^Department of Biochemistry, Faculty of Science, Mahidol University, 272 Rama VI Road, Phayathai, Rajdhevi, Bangkok 10400, Thailand; ^2^Department of Surgery, Rajavithi Hospital, Bangkok 10400, Thailand; ^3^Proteomics Research Laboratory, Genome Institute, National Center for Genetic Engineering and Biotechnology, NSTDA, Pathumthani 12120, Thailand

## Abstract

*Background and Aim*. Cholangiocarcinoma (CCA) is the most frequent biliary malignancy, which poses high mortality rate due to lack of early detection. Hence, most CCA cases are present at the advanced to late stages with local or distant metastasis at the time of diagnosis. Currently available tumor markers including CA19-9 and CEA are inefficient and of limited usage due to low sensitivity and specificity. Here, we attempt to identify serum tumor markers for CCA that can effectively distinguish CCA from benign biliary tract diseases (BBTDs). *Methods*. Serum samples from 19 CCA patients and 17 BBTDs were separated by SDS-PAGE followed with LC-MS/MS and were subjected to statistical analysis and cross-validation to identify proteins whose abundance was significantly elevated or suppressed in CCA samples compared to BBTDs. *Results*. In addition to identifying several proteins previously known to be differentially expressed in CCA and BBTDs, we also discovered a number of molecules that were previously not associated with CCA. These included FAM19A5, MAGED4B, KIAA0321, RBAK, and UPF3B. *Conclusions*. Novel serum biomarkers to distinguish CCA from BBTDs were identified using a proteomic approach. Further validation of these proteins has the potential to provide a biomarker for differentiating CCA from BBTDs.

## 1. Introduction

Cholangiocarcinoma (CCA) is one of the highly aggressive malignant tumors that arise from the cholangiocytes lining biliary trees [1]. The incidence and mortality of the disease continue to increase worldwide, and the highest incidence has been observed in the Southeast Asia, especially in Thailand [2, 3]. The prognosis of this malignancy is poor due to its silent clinical characteristics, difficulties in early diagnosis, and limited therapeutic measures. At present, radiotherapy and chemotherapy do not significantly improve the survival rate, while the resection of detected tumors at the early stage offers the best curative treatment [4]. Clinical presentations of most CCA patients include biliary tract obstruction; however, many cases of benign biliary tract diseases (BBTDs) are also presented with similar clinical symptoms [5]. Differences in the treatment and prognosis between CCA and BBTDs urge us a need to identify accurate tumor biomarkers that can differentially diagnose the CCA from BBTDs. As CCA typically grows along the bile duct without protruding outward as a forming mass, therefore current imaging techniques including ultrasound, computed tomography (CT), and magnetic resonance imaging (MRI) are not efficient to reveal this lesion [6]. Laboratory assessments for CCA are often not sensitive, nor specific enough. Distinguishing between benign and malignant causes of biliary tract obstruction based on biopsies is rather difficult and usually inadequate to provide an accurate measure. Currently, determination of the serum marker carbohydrate antigen 19-9 (CA19-9) concentration is routinely applied in most laboratories for CCA detection. However, a wide range of sensitivity (50–90%) and specificity (54–98%) of this biomarker for CAA has been reported [7–9], and the elevated serum CA19-9 has also been observed in patients with BBTDs [10, 11]; therefore, the use of CA19-9 for differentiating CCA and BBTDs is not reliable. Other serum markers including carcinoembryogenic antigen (CEA) and cancer antigen 125 (CA125) have also been used for detecting CCA, but these markers are not satisfactory for CCA detection due to low specificity and sensitivity for screening [12–14]. Hence, identification of new tumor markers in the serum would be beneficial in the clinical management of this disease.

In recent years, quantitative proteomics has gained considerable attention and investment in order to identify diagnostic biomarkers for several diseases, including a variety of cancers [15]. In the present study, the proteome of serum samples from CCA patients were quantitatively compared with that of patients with BBTDs, who have shared many molecular and imaging features with CCA. A large-scale quantitative global protein profiling of serum coupled with bioinformatic analyses would identify a proteomic signature for effectively differentiating CCA from BBTDs. Patterns of differentially serum protein expression between CCA and BBTD patients were exploited for development of diagnostic or prognostic tool for this type of cancer.

## 2. Methods

### 2.1. Serum Samples

Serum samples were collected from obstructive jaundice patients who underwent endoscopic retrograde cholangiography (ERCP) or biliary tract surgery at Rajavithi Hospital. The use of human materials was approved by the research ethics committee of Rajavithi Hospital. Seventeen patients with BBTDs and 19 CCA patients were enrolled in this study. The diagnosis of CCA was carried out using one of the following criteria: (i) tissue biopsy; (ii) cytology plus radiological (CT scan or MRI) and clinical observation to identify tumor progression at a follow-up of at least two months. Serum samples from these patients were separated by centrifugation and stored at −80°C within 1 h. The biochemical determinations of serum markers, including CEA and CA19-9, were performed using routine automated methods in the Pathological Laboratory at Rajavithi Hospital.

### 2.2. Sample Preparation, Electrophoresis, and Trypsin Digestion

Samples were treated with protease inhibitor cocktail and protein extraction from serum was carried out in lysis buffer containing 8 M urea and 10 mM dithiothreitol (DTT). Protein concentration was determined using Bradford protein assay with bovine serum albumin as a standard. Fifty micrograms of total serum proteins were resolved on 12.5% SDS-PAGE. The gel was then fixed for 30 min in a fixing solution containing 50% methanol, 12% acetic acid, and 0.05% formaldehyde, washed twice for 20 min in 35% ethanol, and then sensitized in 0.02% (w/v) sodium thiosulfate for 2 min with mild agitation. After washing twice for 5 min each with deionized water, the gel was then stained with 0.2% (w/v) silver nitrate for 20 min and washed twice prior to the detection in a developing solution (6% (w/v) sodium carbonate, 0.02% (w/v) sodium thiosulfate and 0.05% formalin). The staining was stopped by incubation in 1.5% Na_2_ EDTA solution for 20 min. Finally, the stained gel was washed three times for 5 min each with deionized water. The gel was scanned using a GS-710 scanner (Bio-Rad, Benicia, CA) before being stored in 0.1% acetic acid until in-gel tryptic digestion.

The gel lanes were divided into 5 fractions according to the standard protein markers and then subdivided into 15 ranges. Each gel range was chopped into pieces (1 mm^3^/piece), which were dehydrated in 100% acetonitrile (ACN) for 5 min with agitation and dried at room temperature for 15 min. Subsequently, the cysteine residues were blocked with 10 mM DTT in 10 mM NH_4_HCO_3_ for 1 h at room temperature and alkylated with 100 mM iodoacetamide in 10 mM NH_4_HCO_3_ for 1 h at room temperature in the dark. The gel pieces were dehydrated twice in 100% ACN for 5 min and then were incubated with 0.20 *μ*g trypsin in 50% ACN/10 mM NH_4_HCO_3_ for 20 min. Purified peptide fractions were dried and reconstituted in 2% ACN and 0.1% formic acid for subsequent LC-MS/MS.

### 2.3. Liquid Chromatography-Tandem Mass Spectrometry (LC/MS-MS)

The LC-MS/MS analysis was carried out using a Waters nanoACQUITY ultra performance liquid chromatography coupled with a SYNAPT HDMS mass spectrometer. A 5-*μ*L aliquot of peptide fractions was injected using a built-in nanoACQUITY auto sampler onto a Symmetry C18 trapping column (200 *μ*m × 180 mm, 5 *μ*m particle size; Waters) at 10 *μ*L/min flow rate for on-line desalting and then separated on a C-18 RP nano-BEH column (75 *μ*m id × 200 mm, 1.7 *μ*m particle size, Waters) and eluted in a 30 min gradient of 2% to 40% ACN in 0.1% formic acid (FA) at 350 nL/min, followed by a 10-min ramping to 80% ACN-0.1% FA and a 5-min holding at 80% ACN-0.1% FA. The column was reequilibrated with 2% ACN-0.1% FA for 20 min prior to the next run. The MS nanoion source contained a 10-*μ*m analyte emitter (New Objective, Woburn, MA) and an additional 20-*μ*m reference sprayer through which a solution of 200 fmol/*μ*L Glu Fibrinopeptide B (Glufib) in 25% ACN-0.1% FA was constantly infused at 200 nL/min for external lock mass correction at 30 s intervals. For all measurements, the MS instrument was operated in V mode (at 10,000 resolution) with positive nanoES ion mode. The instrument was tuned and calibrated by infusion of 200-fmol/*μ*L Glufib and set up for a spray voltage at 2.7 kV and sample cone voltage at 45 eV. The spectral acquisition time was 0.6 sec. In MS expression mode, low energy of trap was set at a constant collision energy of 6 V. In elevated energy of MS expression mode, the collision energy of trap was ramped from 15 to 40 V during each 0.6-s data collection cycle with one complete cycle of low and elevated energy. In transfer collision energy control, 4 V and 7 V were set for low and high energy, respectively. The quadrupole mass analyzer was adjusted such that ions from m/z 200 to 1990 were efficiently transmitted.

### 2.4. Data Processing, Protein Identification, and Data Analysis

Continuum LC-MS data were processed using ProteinLynx Global Server version 2.4 (Waters) for ion detection, clustering, and mass correction. Protein identification was performed with the embedded ion accounting algorithm against NCBI human protein database with the minimum cutoffs of two peptides/proteins. The relative quantitation ratios were log_2_-transformed, processed with median normalization for each sample and rank normalization across the data set. The data were subjected to a 6-fold cross-validation. A differentially expressed (DE) protein was defined as having a *P* value of <0.01, based on *t*-distribution with Welch approximation, in all data sets in the fold validation. The visualization and statistical analyses were performed using the MultiExperiment Viewer (MeV) in the TM4 suite software [16]. Other information including protein categorization and biological function was analyzed according to protein analysis through evolutionary relationships (Panther) protein classification [17]. Known and predicted functional interaction networks of identified proteins were derived from the STRING database version 9.1 [18].

### 2.5. Statistical Analysis

Comparisons between the quantitative variables were performed using either the Mann-Whitney *U* or Student's *t*-test, where appropriate. Qualitative variables were reported as counts, and comparisons between independent groups were performed using Pearson Chi-squared tests. *P* values of less than 0.05 were considered significant.

## 3. Results

### 3.1. Patient Characteristics

A total of 36 subjects were included in this serum proteome study, of which 17 were diagnosed as having BBTDs and 19 were diagnosed as having CCA. The BBTD cases included intrahepatic duct stones, common bile duct stones, and benign bile duct strictures. The CCA cases included perihilar cholangiocarcinoma, intrahepatic cholangiocarcinoma, and middle and distal common bile duct cancer. The clinical characteristics of the patients in this study are shown in Table 1. No statistically significant differences were found among the data of the BBTD patients and those with CCA regarding gender, age, and CEA. Although the level of CA19-9 in the serum of patients with CCA was significantly higher when compared to the control patients, the range of detection in both groups was exactly the same (0.60–10000).

### 3.2. Serum Proteome Profiling

**Figure 1:**
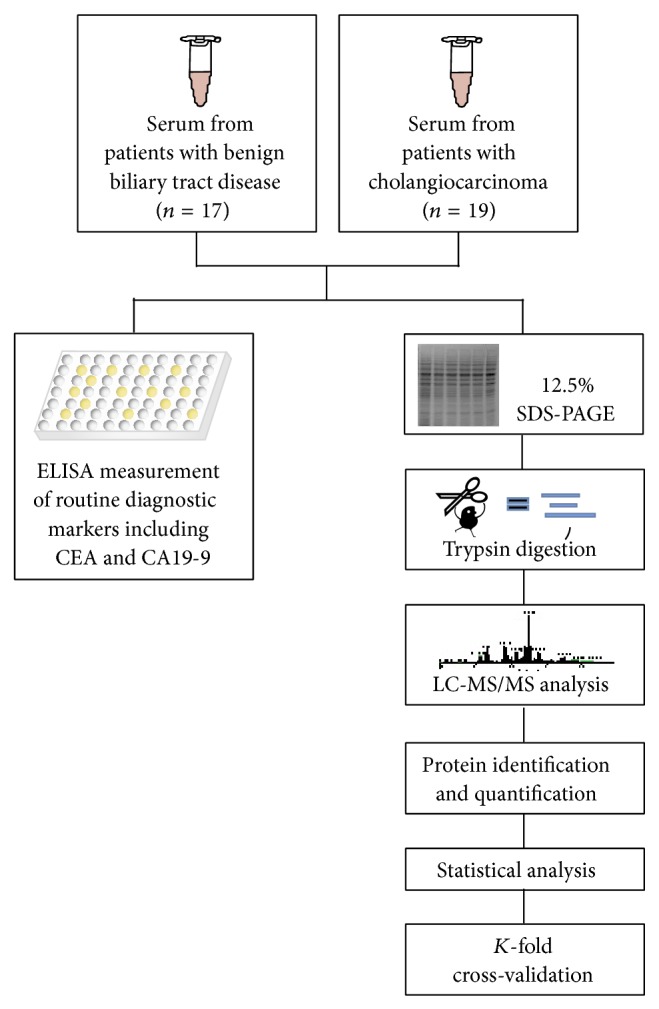
Schematic diagram of the experimental workflow. Serum samples were collected from 17 BBTD patients and 19 CCA patients, which were then subjected to routine ELISA for CEA and CA19-9. Purified proteins from these samples were then separated by SDS-PAGE. After migration, entire lanes were divided into 5 sections, which were excised into slices and treated with in-gel digestion. The resulting tryptic peptides were subjected to reverse-phase LC-MS/MS, from which the mass spectrometric results were then analyzed for protein identification and quantification. The relative quantitation ratios were subjected to statistical analyses and 6-fold cross-validation to retrieve the DE proteins between BBTDs and CCA.

An overview of the experimental strategy conducted in this study is shown in Figure 1. The proteome of serum samples from CCA patients was compared with the serum proteome of the BBTD controls in order to identify the proteins in serum, in particular those that are secreted or leaked from tissues including potential differential protein biomarkers from tumor cells. A total of 951 proteins were identified in all samples. Among these, the ones with altered expression levels in the serum of CCA patients compared to those of BBTD patients were identified. To reduce the effect of biological and experimental variations and the possibility of false-positive protein identification, 6-fold cross-validations were performed. In each fold, BBTD and CCA samples were randomly split into a training set (30 cases with 13–15 BBTD and 15–17 CCA) and an independent validation set (6 cases with 2–4 BBTD and 2–4 CCA). Only proteins identified and quantifiable in all folds in cross-validation were further analyzed, allowing for stringent and sensitive protein identification and quantification of differential proteins.

### 3.3. Identification of Differentially Expressed Proteins between CCA and BBTDs

**Figure 2:**
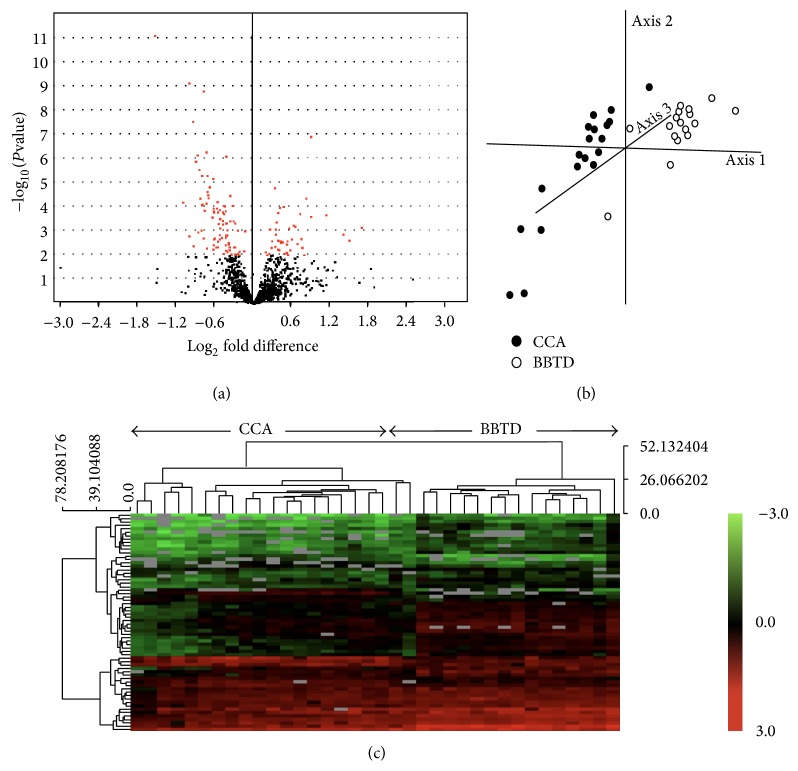
An overview of the DE proteins between BBTDs and CCA. (a) Volcano plots on log_2_ fold change and probability values between BBTDs and CCA cases. Red dots correspond to the identified DE proteins that were cut off at *P* < 0.01. (b) Principle component analysis for DE proteins. Three-dimensional scatter plot represents two clusters of BBTDs and CCA cases based on the DE proteins. Each dot represents a patient with either BBTDs or CCA, as indicated. (c) A hierarchical clustering analysis was carried out on the basis of the expression pattern. The DE proteins were linked together according to their expression (dendrogram on left). BBTD and CCA patients were also clustered (dendrogram on top). The protein-expression intensities were standardized between −3.0 (green) and 3.0 (red).

Applying a *P* value cutoff of <0.01 yielded a total of 94 candidate proteins, with 32 of them up and 62 down in observed abundance for the serum samples from CCA patients comparing to the BBTD controls (Table 2 and Figure 2(a)). We also tested the discriminatory power of these differentially expressed proteins using unsupervised hierarchical clustering. As shown in Figure 2(c), the spectral counts for these proteins resulted in near complete separation of the CCA cases from the BBTD control cases with only two exceptions where BBTD cases were clustered with the CCA samples. However, the PCA scores plot based on the normalized data of serum samples showed a clear separation between the CCA patients and BBTD controls (Figure 2(b)).

**Figure 3:**
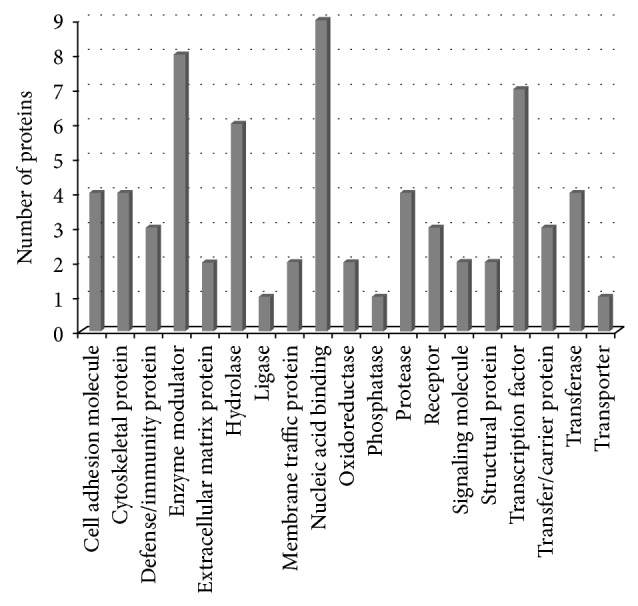
Distribution of DE proteins between BBTDs and CCA according to PANTHER protein classes. The bar chart shows the number of DE proteins in each functional class.

**Figure 4:**
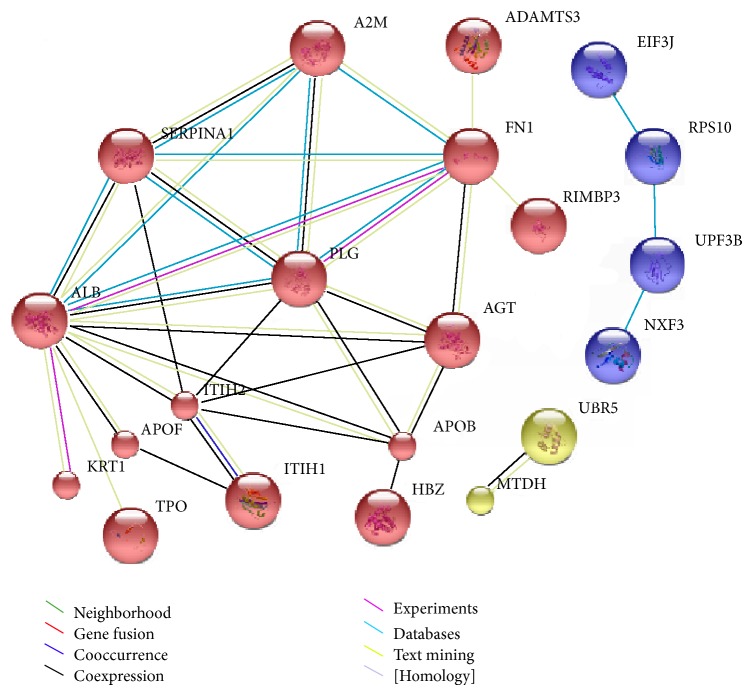
Visualization of protein interaction networks of the DE proteins. Inputting all DE proteins into STRING yielded a network visualizing linkages. The network nodes are proteins, whereas the edges represent the functional associations. Different line colors of edges represent different types of evidence for the association, as indicated in the figure.

The Panther classification system was used to identify the functional attributes of the 94 potential CCA-selective proteins. The analysis of the abundance of each functional category revealed substantial differences in CCA serum proteome compared to the BBTD serum proteome. The number of each functional class of differentially expressed proteins is schematically depicted in Figure 3. The analysis revealed significant enrichment of proteins related to a number of various biological functions such as cell adhesion molecules, cytoskeletal proteins, defense/immunity proteins, enzymes and the modulators, extracellular matrix proteins, membrane traffic proteins, nucleic acid-binding proteins, receptors, signaling molecules, structural proteins, transcription factors, transfer/carrier proteins, and transporters. To gain an overview of the biological interaction among the identified proteins, we also constructed the protein-protein functional networks using String database (Figure 4). The protein network analysis provides us a clearer view of a complex framework of proteins that might result in the differences in CCA and BBTDs.

**Figure 5:**
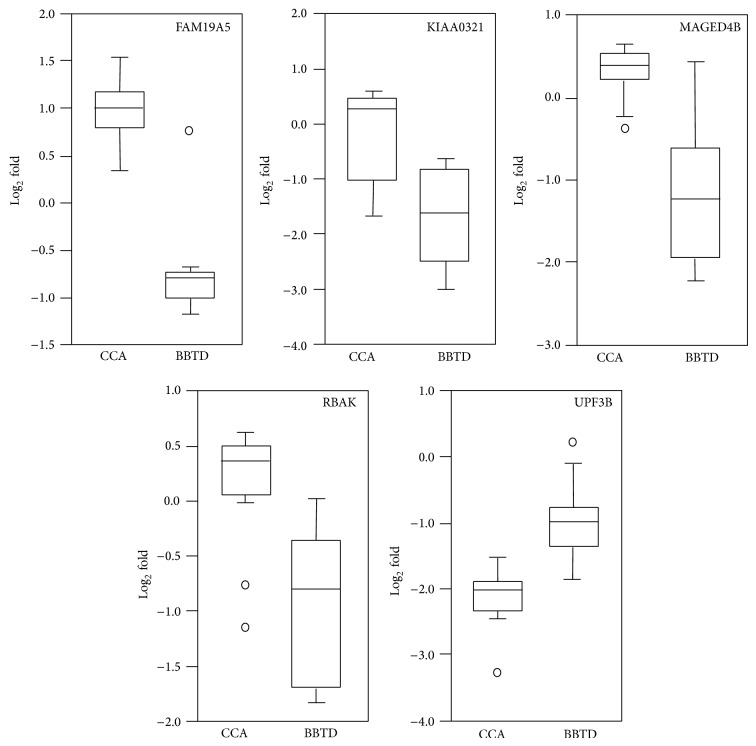
Comparison of the top five differentially expressed proteins between BBTDs and CCA. Normalized log_2_-transformed data were used to create box plots, in which the horizontal lines of each box correspond to the first, second, and third quartiles (25%, 50%, and 75%, resp.) and the whiskers correspond to the maximum and minimum values.

To determine the distinguishing performance of the top five differentially expressed proteins in terms of fold-change, the comparison of the averaged log_2_ folds of family with sequence similarity 19 (chemokine (C-C motif)-like), member A5 (FAM19A5) protein, KIAA0321 protein, melanoma-associated antigen D4 (MAGED4B), RB-associated KRAB zinc finger protein (RBAK), and regulator of nonsense transcripts 3B (UPF3B), between CCA and BBTD cases from all cross-validation cohorts was shown in Figure 5. However, due to the limited resources and the lack of availability of an independent validation set, the diagnostic relevance of such molecules for CCA requires further investigation.

## 4. Discussion

CCA is the second most prevalent primary hepatobiliary malignancy and represents about 3% of all gastrointestinal cancers [1]. It is associated with inflammatory conditions in the biliary system, and patients with risk factors such as primary sclerosing cholangitis and liver fluke infestations have a higher risk for CCA development [1–3]. The generally late clinical presentation of CCA results in a high mortality. At present, the most commonly studied and routinely used serum biomarkers for detecting CCA include CEA and CA19-9 [6]. However, they are nonspecific to CCA and can be elevated in the setting of other gastrointestinal malignancies or other benign conditions, such as cholangitis, cirrhosis, and hepatolithiasis [7–14]. Based on the results in this study, both CEA and CA19-9 could not also distinguish the patients with CCA and BBTDs in our sample cohort as both appeared to be nonspecific for either case. Hence, there is an urgent need for new diagnostic targets. In this study, we evaluated the differential proteome in the serum between the BBTD controls and CCA patients and identified potential biomarker panels to aid in the diagnosis of these common liver diseases.

Total proteins were retrieved from the whole serum without the depletion of high abundant proteins due to the fact that additional steps may not help enrich the level of low abundant proteins and may reduce reproducibility from one sample to the others [19]. Among the identified proteins, we found that a number of them had previously been described in the context of CCA, confirming the validity of our quantitative proteomic approach. These included overexpression of MAGED4 [20] and DNA mismatch repair protein (MLH1) [21, 22], downregulation of albumin (ALB) [20], apolipoprotein B (APOB) [20], apolipoprotein A-II (APOA2) [20], and interalpha (globulin) inhibitor H1 (ITIH1) [20, 23]. Expression of serum alpha 1-macroglobulin (A2M) was found to be significantly higher in BBTD compared to CCA patients. Consistently, it has also been reported that the serum A2M increased in patients with liver malignancies including CCA but markedly elevated in hepatic cirrhosis [24]. Fibronectin 1 (FN1) in serum of CCA patients seemed to be lower than that of BBTD patients. Biliary FN1 has been reported as a differential biomarker of benign and malignant diseases [25]. Similarly, serum plasminogen (PLG) of CCA cases was significantly lower than that of BBTD controls. PLG in malignant livers including CCA has been demonstrated to be lower than that of the cirrhosis patients [26]. Other serum proteins were also found differentially expressed between CCA and BBTD including angiotensinogen (AGT), ADAM metallopeptidase with thrombospondin type 1 motif 3 (ADAMTS3), hemoglobin, zeta (HBZ), keratin-1 (KRT1), keratin-10 (KRT10), and serpin peptidase inhibitor, clade A (alpha-1 antiproteinase, antitrypsin), and member 1 (SERPINA1). However, the validation of these identified proteins is needed in order to determine if they can be clinically useful as differential biomarkers for CCA and BBTD.

The top five proteins which exhibited the maximal fold change between CCA and BBTD consisted of FAM19A5, MAGED4B, KIAA0321, RBAK, and UPF3B. FAM19A5 belongs to the TAFA family of small secreted proteins, which are brain-specific and distantly related to MIP-1 alpha, a member of the CC-chemokine family [27]. This family of proteins has been postulated to function as brain-specific chemokines or neurokines that act as regulators of immune and nervous cells, although the association of this protein and CCA pathogenesis has yet to be evaluated. For MAGED4B, its overexpression has been linked to malignant tumors and poor patient outcome in many types of cancer including breast [28], oral squamous cell carcinoma [29], and hepatocellular carcinoma [30]. However, there are no data available on the expression and the diagnostic or prognostic relevance of MAGED4B in CCA and BBTDs. KIAA0321 is a zinc finger FYVE domain-containing protein, which mediates binding of these proteins to membrane lipids and may be involved in the abscission step of cytokinesis. However, the relevance of this protein and cancer development is yet to be elucidated [31]. RBAK is a member of a known family of transcriptional repressors that contain zinc fingers of the Kruppel type, which interacts with the tumor suppressor retinoblastoma 1. It has been shown that RBAK is expressed ectopically in human fibroblast cells [32]. Since fibroblasts in the stroma of desmoplastic cancers provide optimal microenvironment for CCA progression and they usually become susceptible for apoptosis [33], it would therefore be possible that overexpression of serum RBAK in CCA patients may be from apoptogenic cancer-associated fibroblasts. UPF3B has been reported to be overexpressed in the patients with alcoholic hepatitis [34], but there is currently no link on UPF3B and cancer yet.

In conclusion we identified proteins in the serum that can potentially discriminate patients with CCA from BBTD individuals through proteomic approach using highly stringent analysis with cross-validation. These proteins will be clinically useful to prevent misdiagnosis between CCA and BBTD as they have similar clinical symptoms. Further independent validation of these biomarkers is certainly required using greater numbers of samples from patients with CCA and a wider range of BBTD conditions to test its robustness and obtain the ones with the greatest diagnostic power for differentiating patients with CCA from BBTD controls.

## Figures and Tables

**Table 1:** Clinical characteristics of patients with benign biliary tract diseases (BBTDs) and cholangiocarcinoma (CCA) in this study.

Characteristics	BBTDs	CCA	*P* values
Number of patients	17	19	—
Sex			
(Male : female)	10 : 7	10 : 9	0.085
Age (years)			
Mean ± S.D.	52.6 ± 13	60.9 ± 13	0.285
CEA (U/mL)			
Median	8.70	9.87	0.711
(Min–max)	(0.62–118.30)	(1.47–410.40)	
CA19-9 (ng/mL)			
Median	48.03	4355.50	0.015
(Min–max)	(0.60–10000)	(0.60–10000)	

**Table 2:** A list of differentially expressed serum proteins between CCA and BBTDs. The protein expression measurements were averaged and represented as log_2_-transformed intensity values with standard deviation. The *P* values are also indicated.

Protein names	ID details	GI accession	CCA mean ± SD	BBTD mean ± SD	*P* values
CCA > BBTD					
ABHD11	Alpha/beta hydrolase domain-containing protein 11	23200008	1.20 ± 0.4	0.82 ± 0.3	0.002
—	Antioxidized LDL immunoglobulin light chain variable region	62868476	0.88 ± 0.3	0.55 ± 0.3	0.004
—	Chain L, crystal structure of the Fab fragment of nimotuzumab. An antiepidermal growth factor receptor antibody	255311843	1.93 ± 0.2	1.72 ± 0.3	0.010
—	Complement factor H	758073	−0.86 ± 0.3	−1.51 ± 0.5	0.001
COG7	Conserved oligomeric Golgi complex subunit 7	23957690	−0.50 ± 0.4	−1.23 ± 0.5	<0.001
DHDDS	Dehydrodolichyl diphosphate synthase	13177736	0.14 ± 0.6	−0.61 ± 0.5	0.005
MLH1	DNA mismatch repair protein	4557757	−0.49 ± 0.5	−1.00 ± 0.5	0.004
EIF3J	Eukaryotic translation initiation factor 3 subunit J	83281438	2.40 ± 0.4	1.95 ± 0.4	0.002
FAM19A5	FAM19A5 protein	71052198	0.98 ± 0.3	−0.69 ± 0.7	<0.001
HBZ	Hemoglobin subunite zeta	4885397	−0.23 ± 0.3	−0.78 ± 0.4	0.001
V4-34	IgG	2632200	0.54 ± 0.4	0.20 ± 0.2	0.001
—	Immunoglobulin heavy chain variable region	37694587	1.46 ± 0.4	0.93 ± 0.5	0.003
IGK	Immunoglobulin kappa light chain VLJ region	21669309	1.98 ± 0.3	1.68 ± 0.1	<0.001
IL16	Interleukin 16	119619506	0.15 ± 0.4	−0.36 ± 0.6	0.008
KIAA0321	KIAA0321 protein	2224583	−0.25 ± 0.8	−1.70 ± 0.9	0.002
KIAA0612	KIAA0612 protein	34327964	−1.17 ± 0.4	−1.76 ± 0.5	0.006
KIAA0896	KIAA0896 protein	71891755	0.18 ± 0.6	−0.50 ± 0.6	0.003
MAGED4B	Melanoma-associated antigen D4	29337296	0.33 ± 0.3	−1.17 ± 0.9	0.002
NXF3	Nuclear RNA export factor 3	11545757	0.59 ± 0.4	−0.29 ± 0.5	<0.001
PAXBP1	PAX3- and PAX7-binding protein 1	22035565	0.64 ± 0.1	0.39 ± 0.2	0.001
LOC390791	Peptidyl-prolyl cis-trans isomerase A-like	310113085	−0.50 ± 0.3	−1.24 ± 0.4	0.002
PLEKHO2	Pleckstrin homology domain-containing family O member 2	33457316	−0.29 ± 0.4	−0.91 ± 0.4	0.001
PLEKHM2	PLEKHM2 protein	26251859	−0.46 ± 0.4	−1.06 ± 0.5	0.002
RBAK	RB-associated KRAB zinc finger protein	13430850	0.17 ± 0.5	−0.96 ± 0.7	<0.001
PTPRG	Receptor tyrosine phosphatase gamma	1263069	0.90 ± 0.3	0.54 ± 0.2	<0.001
RPS10	Ribosomal protein S10	3088338	0.99 ± 0.2	0.67 ± 0.1	<0.001
NOB1	RNA-binding protein NOB1	7661532	−0.81 ± 0.4	−1.26 ± 0.4	0.002
VAT1	Synaptic vesicle membrane protein VAT-1	18379349	0.57 ± 0.4	0.15 ± 0.3	0.003
TRAC-1	T3 receptor-associating cofactor-1	1911770	0.12 ± 0.3	−0.28 ± 0.2	<0.001
—	Unnamed protein product	10433849	1.67 ± 0.5	0.77 ± 0.3	<0.001
—	Unnamed protein product	21752201	0.27 ± 0.2	−0.07 ± 0.3	0.001
CCA < BBTD					
GCAT	2-Amino-3-ketobutyrate coenzyme A ligase	7657118	−1.62 ± 0.5	−1.17 ± 0.3	0.002
ALB	Albumin	119626083	1.05 ± 0.3	1.45 ± 0.3	<0.001
SERPINA1	Alpha-1-antitrypsin	1703025	0.84 ± 0.4	1.24 ± 0.3	<0.001
A2M	Alpha-2-macroglobulin	177872	−0.04 ± 0.4	0.52 ± 0.3	<0.001
AGT	Angiotensinogen	4261988	0.09 ± 0.6	0.79 ± 0.2	<0.001
apo AII	Apolipoprotein	671882	0.04 ± 0.3	0.31 ± 0.2	0.003
APOB	Apolipoprotein B-100	105990532	−1.67 ± 0.4	−0.86 ± 0.5	<0.001
ARID5B	AT-rich interactive domain-containing protein 5B	74136549	0.08 ± 0.5	0.71 ± 0.7	0.006
BAZ2	BWSCR2 associated zinc-finger protein BAZ2	6002480	−0.50 ± 0.9	0.23 ± 0.3	0.005
C1orf87	C1orf87 protein	27503780	−0.91 ± 0.4	−0.49 ± 0.3	0.001
PDEA	cGMP phosphodiesterase	2366987	0.11 ± 0.4	0.73 ± 0.3	<0.001
	Chain D, The Nucleosome Containing A Testis-Specific Histone Variant	296863399	−2.56 ± 0.4	−2.04 ± 0.5	0.002
C4A	Complement C4-A	476007827	−0.81 ± 0.7	−0.02 ± 0.3	<0.001
DCAF15	DDB1- and CUL4-associated factor 15	78486540	0.87 ± 0.4	1.31 ± 0.2	<0.001
FN1	Fibronectin 1	53791223	−0.98 ± 0.5	−0.29 ± 0.4	<0.001
FLJ00044	FLJ00044 protein	10440418	−1.02 ± 0.4	−0.59 ± 0.2	0.004
FLJ16008	FLJ16008 protein, isoform CRA_b	119615716	1.79 ± 0.1	1.94 ± 0.2	0.006
GNG5	Guanine nucleotide-binding protein G(I)/G(S)/G(O) subunit gamma-5	4885287	0.94 ± 0.3	1.24 ± 0.1	<0.001
hCG_1817987	hCG1817987	119612015	−0.09 ± 0.3	0.36 ± 0.3	<0.001
hCG_1981701	hCG1981701	119572460	−1.14 ± 0.5	−0.52 ± 0.5	0.002
hCG_2008076	hCG2008076	119592316	0.05 ± 0.4	0.42 ± 0.2	0.012
hCG_2008267	hCG2008267	119592800	−0.07 ± 0.4	0.35 ± 0.2	0.001
hCG_201157	hCG201157	119576573	0.64 ± 0.3	0.89 ± 0.3	0.009
hCG_2020343	hCG2020343	119629275	−0.95 ± 0.4	−0.27 ± 0.4	<0.001
—	Hypothetical protein	12224988	−1.46 ± 0.5	−0.90 ± 0.4	<0.001
FLJ22688	Hypothetical protein FLJ22688, isoform CRA_b	119572924	−0.93 ± 0.6	−0.17 ± 0.4	<0.001
LOC286076	Hypothetical protein LOC286076	119602615	1.19 ± 0.4	1.52 ± 0.2	0.003
IgA1	Ig Aalpha1 Bur	223099	1.84 ± 0.6	2.28 ± 0.3	0.006
	Immunoglobulin heavy chain variable region	37694587	1.48 ± 0.3	1.70 ± 0.1	0.007
ITIH1	Interalpha (globulin) inhibitor H1	825681	−0.74 ± 0.4	0.08 ± 0.6	0.001
ITIH2	Interalpha (globulin) inhibitor H2	119606784	−1.44 ± 0.5	−0.54 ± 0.4	<0.001
KRT1	Keratin 1	11935049	−1.12 ± 0.3	−0.56 ± 0.4	<0.001
KRT10	Keratin-10	307086	−2.40 ± 0.5	−1.50 ± 0.6	<0.001
KIAA0366	KIAA0366 protein	2224673	−1.45 ± 0.4	−0.61 ± 0.6	<0.001
KIAA0920	KIAA0920 protein	40788986	1.05 ± 0.3	1.37 ± 0.2	<0.001
KIAA1234	KIAA1234 protein	6330736	−0.70 ± 0.4	−0.38 ± 0.3	0.007
KIAA1529	KIAA1529 protein	7959325	1.33 ± 0.3	2.38 ± 0.4	<0.001
MAGEB2	Melanoma-associated antigen B2	222418639	−0.48 ± 0.3	−0.07 ± 0.4	0.001
MTDH	Metadherin	119612168	−0.45 ± 0.3	−0.09 ± 0.3	<0.001
MUC16	Mucin-16	74716283	−0.49 ± 0.5	0.41 ± 0.4	<0.001
MYOT	Myotilin	5803106	−0.41 ± 0.5	0.30 ± 0.3	<0.001
NPTX1	Neuronal pentraxin 1	1438954	1.51 ± 0.3	1.74 ± 0.2	0.010
PLG	Plasminogen	38051823	1.67 ± 0.3	2.17 ± 0.4	<0.001
GALNT2	Polypeptide N-acetylgalactosaminyltransferase 2	4758412	−0.99 ± 0.3	−0.50 ± 0.3	<0.001
FAM83E	Protein FAM83E	153251792	0.70 ± 0.5	1.33 ± 0.3	0.006
LOC100131107	Putative UPF0607 protein ENSP00000383783	239741331	1.03 ± 0.7	2.07 ± 0.7	0.002
RAB-R	RAB-R protein	4102709	−0.83 ± 0.4	−0.05 ± 0.3	0.002
UPF3B	Regulator of nonsense transcripts 3B	18375528	−2.17 ± 0.5	−0.99 ± 0.6	<0.001
RIMBP3	RIMBP3 protein	71052030	0.07 ± 0.4	0.69 ± 0.4	<0.001
	Suppressor of cytokine signaling 3	54695958	0.85 ± 0.4	1.23 ± 0.2	0.004
	Testis specific kinase-1	21886788	−1.65 ± 0.3	−1.07 ± 0.7	0.005
TTC34	Tetratricopeptide repeat protein 34	239741018	−0.54 ± 0.5	0.34 ± 0.3	<0.001
TPO	Thyroid peroxidase	4680721	0.69 ± 0.4	1.15 ± 0.2	<0.001
—	Ubiquitously transcribed tetratricopeptide repeat protein Y-linked transcript	148733192	−1.43 ± 0.4	−0.88 ± 0.5	0.001
—	Unnamed protein product	34531956	0.84 ± 0.3	1.17 ± 0.2	<0.001
—	Unnamed protein product	10435479	0.76 ± 0.4	1.15 ± 0.3	0.003
—	Unnamed protein product	194384842	1.24 ± 0.3	1.67 ± 0.2	<0.001
—	Unnamed protein product	22760231	1.14 ± 0.5	1.77 ± 0.3	<0.001
—	Unnamed protein product	194381130	−2.08 ± 0.4	−1.34 ± 0.5	<0.001
DBP	Vitamin D-binding protein	455970	0.88 ± 0.5	1.30 ± 0.3	0.007
ZNF410	Zinc finger protein 410	119601547	−0.27 ± 0.4	0.46 ± 0.3	<0.001
ZnF_RBZ	ZIS1	4191327	0.03 ± 0.4	0.55 ± 0.4	0.001

## References

[1] Blechacz B., Gores G. J. (2008). Cholangiocarcinoma: advances in pathogenesis, diagnosis, and treatment. *Hepatology*.

[2] Shaib Y., El-Serag H. B. (2004). The epidemiology of cholangiocarcinoma. *Seminars in Liver Disease*.

[3] Sripa B., Pairojkul C. (2008). Cholangiocarcinoma: lessons from Thailand. *Current Opinion in Gastroenterology*.

[4] Nakagohri T., Kinoshita T., Konishi M., Takahashi S., Gotohda N. (2008). Surgical outcome and prognostic factors in intrahepatic cholangiocarcinoma. *World Journal of Surgery*.

[5] Deng F.-T., Li Y.-X., Ye L., Tong L., Yang X.-P., Chai X.-Q. (2010). Hilar inflammatory pseudotumor mimicking hilar cholangiocarcinoma. *Hepatobiliary & Pancreatic Diseases International*.

[6] Van Beers B. E. (2008). Diagnosis of cholangiocarcinoma. *HPB*.

[7] Patel A. H., Harnois D. M., Klee G. G., Larusso N. F., Gores G. J. (2000). The utility of CA 19-9 in the diagnoses of cholangiocarcinoma in patients without primary sclerosing cholangitis. *The American Journal of Gastroenterology*.

[8] Qin X.-L., Wang Z.-R., Shi J.-S., Lu M., Wang L., He Q.-R. (2004). Utility of serum CA19-9 in diagnosis of cholangiocarcinoma: in comparison with CEA. *World Journal of Gastroenterology*.

[9] Levy C., Lymp J., Angulo P., Gores G. J., Larusso N., Lindor K. D. (2005). The value of serum CA 19-9 in predicting cholangiocarcinomas in patients with primary sclerosing cholangitis. *Digestive Diseases and Sciences*.

[10] Ong S. L., Sachdeva A., Garcea G. (2008). Elevation of carbohydrate antigen 19.9 in benign hepatobiliary conditions and its correlation with serum bilirubin concentration. *Digestive Diseases and Sciences*.

[11] Principe A., Del Gaudio M., Grazi G. L., Paolucci U., Cavallari A. (2003). Mirizzi syndrome with cholecysto-choledocal fistula with a high CA19-9 level mimicking biliary malignancies: a case report. *Hepato-Gastroenterology*.

[12] Nakanuma Y., Sasaki M. (1989). Expression of blood group-related antigens in the intrahepatic biliary tree and hepatocytes in normal livers and various hepatobiliary diseases. *Hepatology*.

[13] Nakeeb A., Lipsett P. A., Lillemoe K. D. (1996). Biliary carcinoembryonic antigen levels are a marker for cholangiocarcinoma. *The American Journal of Surgery*.

[14] Chen C.-Y., Shiesh S.-C., Tsao H.-C., Lin X.-Z. (2002). The assessment of biliary CA 125, CA 19-9 and CEA in diagnosing cholangiocarcinoma—the influence of sampling time and hepatolithiasis. *Hepato-Gastroenterology*.

[15] Zhao Y., Lee W.-N. P., Xiao G. G. (2009). Quantitative proteomics and biomarker discovery in human cancer. *Expert Review of Proteomics*.

[16] Saeed A. I., Bhagabati N. K., Braisted J. C. (2006). TM4 microarray software suite. *Methods in Enzymology*.

[17] Mi H., Muruganujan A., Casagrande J. T., Thomas P. D. (2013). Large-scale gene function analysis with the PANTHER classification system. *Nature Protocols*.

[18] Franceschini A., Szklarczyk D., Frankild S. (2013). STRING v9.1: protein-protein interaction networks, with increased coverage and integration. *Nucleic Acids Research*.

[19] Bruix J., Llovet J. M. (2002). Prognostic prediction and treatment strategy in hepatocellular carcinoma. *Hepatology*.

[20] Chen M.-H., Lin K.-J., Yang W.-L. R. (2013). Gene expression-based chemical genomics identifies heat-shock protein 90 inhibitors as potential therapeutic drugs in cholangiocarcinoma. *Cancer*.

[21] Limpaiboon T., Khaenam P., Chinnasri P. (2005). Promoter hypermethylation is a major event of *hMLH1* gene inactivation in liver fluke related cholangiocarcinoma. *Cancer Letters*.

[22] Liengswangwong U., Karalak A., Morishita Y. (2006). Immunohistochemical expression of mismatch repair genes: a screening tool for predicting mutator phenotype in liver fluke infection-associated intrahepatic cholangiocarcinoma. *World Journal of Gastroenterology*.

[23] Subrungruang I., Thawornkuno C., Porntip C.-P., Pairojkul C., Wongkham S., Petmitr S. (2013). Gene expression profiling of intrahepatic cholangiocarcinoma. *Asian Pacific Journal of Cancer Prevention*.

[24] Changbumrung S., Migasena P., Supawan V., Juttijudata P., Buavatana T. (1988). Serum protease inhibitors in opisthorchiasis, hepatoma, cholangiocarcinoma, and other liver diseases. *The Southeast Asian Journal of Tropical Medicine and Public Health*.

[25] Chen C.-Y., Lin X.-Z., Tsao H.-C., Shiesh S.-C. (2003). The value of biliary fibronectin for diagnosis of cholangiocarcinoma. *Hepato-Gastroenterology*.

[26] Alkim H., Ayaz S., Sasmaz N., Oguz P., Sahin B. (2012). Hemostatic abnormalities in cirrhosis and tumor-related portal vein thrombosis. *Clinical and Applied Thrombosis/Hemostasis*.

[27] Tang Y. T., Emtage P., Funk W. D. (2004). TAFA: a novel secreted family with conserved cysteine residues and restricted expression in the brain. *Genomics*.

[28] Germano S., Kennedy S., Rani S. (2012). MAGE-D4B is a novel marker of poor prognosis and potential therapeutic target involved in breast cancer tumorigenesis. *International Journal of Cancer*.

[29] Chong C. E., Lim K. P., Gan C. P. (2012). Over-expression of MAGED4B increases cell migration and growth in oral squamous cell carcinoma and is associated with poor disease outcome. *Cancer Letters*.

[30] Takami H., Kanda M., Oya H. (2013). Evaluation of MAGE-D4 expression in hepatocellular carcinoma in Japanese patients. *Journal of Surgical Oncology*.

[31] Kutateladze T. G. (2006). Phosphatidylinositol 3-phosphate recognition and membrane docking by the FYVE domain. *Biochimica et Biophysica Acta—Molecular and Cell Biology of Lipids*.

[32] Skapek S. X., Jansen D., Wei T.-F. (2000). Cloning and characterization of a novel Kruppel-associated box family transcriptional repressor that interacts with the retinoblastoma gene product, RB. *The Journal of Biological Chemistry*.

[33] Mertens J. C., Fingas C. D., Christensen J. D. (2013). Therapeutic effects of deleting cancer-associated fibroblasts in cholangiocarcinoma. *Cancer Research*.

[34] Affò S., Dominguez M., Lozano J. J. (2013). Transcriptome analysis identifies TNF superfamily receptors as potential therapeutic targets in alcoholic hepatitis. *Gut*.

